# Circadian Rhythm Disruptions and Cardiovascular Disease Risk: The Special Role of Melatonin

**DOI:** 10.3390/cimb47080664

**Published:** 2025-08-17

**Authors:** Jarosław Nuszkiewicz, Wojciech Rzepka, Julia Markiel, Marta Porzych, Alina Woźniak, Karolina Szewczyk-Golec

**Affiliations:** 1 Department of Medical Biology and Biochemistry, Faculty of Medicine, Ludwik Rydygier Collegium Medicum in Bydgoszcz, Nicolaus Copernicus University in Toruń, 24 Karłowicza St., 85-092 Bydgoszcz, Poland; karosz@cm.umk.pl; 2 Student Research Club of Medical Biology and Biochemistry, Department of Medical Biology and Biochemistry, Faculty of Medicine, Ludwik Rydygier Collegium Medicum in Bydgoszcz, Nicolaus Copernicus University in Toruń, 24 Karłowicza St., 85-092 Bydgoszcz, Poland; 316679@stud.umk.pl (W.R.); 316560@stud.umk.pl (J.M.); 321536@stud.umk.pl (M.P.)

**Keywords:** cardiovascular disease, chronotherapy, circadian rhythms, melatonin, oxidative stress, shift work

## Abstract

Circadian rhythms are endogenous biological cycles that regulate essential cardiovascular functions, including blood pressure, heart rate, vascular tone, and metabolic homeostasis. Disruption of these rhythms due to factors such as shift work, artificial light at night, irregular sleep–wake cycles, or mistimed eating has been increasingly recognized as an independent risk factor for cardiovascular disease. A growing body of evidence links circadian misalignment to key pathophysiological mechanisms, including endothelial dysfunction, oxidative stress, inflammation, and autonomic imbalance. Melatonin, a hormone produced primarily by the pineal gland, plays a central role in circadian regulation and exhibits potent antioxidant, anti-inflammatory, and cardiometabolic properties. This narrative review synthesizes current findings on the interplay between circadian disruption and cardiovascular risk, with a particular emphasis on the mechanistic and therapeutic role of melatonin. We also highlight the potential of chronotherapeutic strategies, such as timed melatonin supplementation, antihypertensive dosing, and time-restricted eating, to restore circadian alignment and improve cardiovascular outcomes. Despite promising data, translation into clinical practice remains limited. Future research should focus on identifying practical circadian biomarkers, refining chronotherapy protocols, and integrating circadian variables into risk models and clinical workflows.

## 1. Introduction

Circadian rhythms are endogenous, self-sustained oscillations that cycle approximately every 24 h and regulate a broad spectrum of physiological functions, including the sleep–wake cycle, core body temperature, blood pressure (BP), hormone secretion, glucose metabolism, immune responses, and vascular tone [1]. These rhythms are orchestrated by a hierarchical system of molecular clocks, with the suprachiasmatic nucleus (SCN) in the hypothalamus acting as the master pacemaker. The SCN synchronizes peripheral clocks located in nearly every organ and tissue, ensuring temporal coordination across the body [2]. Among all circadian outputs, the sleep–wake cycle is the most overt and well-characterized rhythm, influenced primarily by the light–dark cycle and modulated by neuroendocrine signals such as melatonin [3].

Recent research has underscored that circadian rhythms not only control the timing of physiological functions but also influence their magnitude and responsiveness to external stimuli [4]. For instance, hormonal secretions such as cortisol, insulin, and catecholamines follow circadian patterns that shape metabolic homeostasis and stress responses [1]. Similarly, BP and endothelial tone fluctuations align with predictable daily variations in sympathetic and parasympathetic activity [5]. These tightly regulated temporal patterns help anticipate environmental demands and optimize physiological efficiency. Importantly, misalignment of circadian timing, whether due to behavioral or genetic factors, can lead to decoupling of physiological systems, promoting dysfunction and disease [6].

Melatonin, a neurohormone synthesized primarily by the pineal gland in response to darkness, is one of the key mediators linking the circadian clock to cardiovascular physiology. The secretion of melatonin follows a robust diurnal pattern, with peak concentrations typically occurring between 2:00 and 4:00 a.m. and minimal levels during daytime [7]. This rhythmic profile is driven by light exposure transduced via the retinohypothalamic tract to the SCN, which regulates pineal activity through a multisynaptic sympathetic pathway. Even low-intensity artificial light at night (ALAN) can suppress nocturnal melatonin synthesis, disrupting circadian homeostasis [8].

Melatonin acts as a systemic circadian signal, conveying “darkness” information to tissues and helping synchronize peripheral oscillators with the environmental day–night cycle [9]. Beyond this chronobiotic role, melatonin exhibits a broad range of pleiotropic biological effects relevant to cardiovascular health. It is a potent scavenger of reactive oxygen species (ROS) and reactive nitrogen species (RNS), an upregulator of endogenous antioxidant enzymes such as superoxide dismutases (SODs) and glutathione peroxidases (GPxs) [10], and a modulator of immune and inflammatory responses [11]. Notably, melatonin has been shown to reduce BP, attenuate sympathetic tone, and improve endothelial function in experimental and clinical settings [12].

Given the increasing prevalence of circadian disruption in modern society and the accumulating evidence linking melatonin to cardioprotection, there is growing interest in exploring this molecule as a potential therapeutic agent in cardiovascular prevention. Circadian disruption (also referred to as chronodisruption) describes a misalignment between endogenous biological rhythms and the external environment, often caused by factors such as shift work, irregular sleep–wake schedules, or exposure to light at night [13,14]. While multiple studies have addressed circadian regulation or melatonin systemic effects, few have comprehensively integrated molecular circadian mechanisms, environmental disruption, and melatonin cardioprotective potential within a single conceptual framework [15,16,17]. This article presents a narrative review of the current literature on circadian disruption and cardiovascular risk, with a particular focus on the role of melatonin as both a mechanistic mediator and a potential therapeutic agent. By integrating molecular, clinical, and environmental perspectives, this review aims to provide a comprehensive framework for understanding the chronobiological determinants of cardiovascular health.

We conducted a targeted, narrative search of PubMed, Scopus, and Web of Science databases covering the period from January 2000 to July 2025. The search used combinations of the following terms: “circadian rhythm” OR “circadian disruption” OR “chronodisruption” AND “melatonin” AND (“cardiovascular” OR “hypertension” OR “atherosclerosis” OR “heart rate variability”) AND (“shift work” OR “artificial light at night” OR “time-restricted feeding”). Inclusion criteria were as follows: (i) peer-reviewed articles in English; (ii) human clinical trials, observational studies, or preclinical models with cardiovascular relevance; (iii) review articles that provided a mechanistic or epidemiological synthesis. Exclusion criteria were as follows: (i) non-peer-reviewed material, conference abstracts, or editorials; (ii) studies without a circadian or cardiovascular component; (iii) case reports unless mechanistically informative. Reference lists of key publications and recent meta-analyses were hand-searched to identify additional studies.

## 2. Circadian Rhythms and Cardiovascular Function—Molecular Foundations of Circadian Rhythms

### 2.1. Molecular Basis of Circadian Rhythms

At the cellular level, circadian rhythms are governed by a transcriptional–translational feedback loop (TTFL) composed of core clock genes and proteins that generate rhythmic oscillations in gene expression with an approximately 24 h periodicity. The central components of this loop include the CLOCK (circadian locomotor output cycles kaput) and BMAL1 (brain and muscle aryl hydrocarbon receptor nuclear translocator (Arnt)-like protein 1) proteins, which heterodimerize and bind to enhancer (E)-box elements in the promoter regions of target genes, activating the transcription of *Period* (*PER1*, *PER2*, and *PER3*) and *Cryptochrome* (*CRY1* and *CRY2*) genes [18,19,20,21,22,23]. As PER and CRY proteins accumulate in the cytoplasm, they form inhibitory complexes that translocate into the nucleus and suppress their transcription by interacting with the CLOCK–BMAL1 complex. This negative feedback loop is delayed by time-dependent processes of transcription, translation, and protein degradation, allowing the system to maintain approximately 24 h stable rhythms [18,19,22,24].

This core loop is further regulated by secondary interlocking feedback mechanisms. Nuclear receptors, such as reverse orientation c-erbA gene α (REV-ERBα), known as nuclear receptor subfamily 1 group D member 1 (NR1D1), and retinoic acid receptor-related orphan receptor alpha (RORα), modulate the transcription of BMAL1 itself, forming stabilizing loops that fine-tune the amplitude and phase of oscillations [18,19,21,22,23]. These interconnected circuits collectively establish the molecular architecture of circadian rhythmicity, operating not only in the SCN but also in peripheral tissues, including the heart, vasculature, kidney, and liver. The precise timing of gene expression orchestrated by these mechanisms is crucial for synchronizing metabolic and physiological processes with daily environmental cycles [25].

### 2.2. Blood Pressure Regulation and Peripheral Clocks

The cardiovascular system exhibits robust circadian oscillations that influence a wide range of physiological parameters, including BP, heart rate (HR), vascular tone, and thrombotic potential [26]. BP typically follows a biphasic daily pattern, characterized by a nocturnal decline (“dipping”) during sleep and a marked surge in the early morning upon waking. This daily rhythm reflects the interplay between endogenous circadian regulation and behavioral cues such as physical activity, posture, and feeding cycles [27]. Laboratory protocols that isolate circadian influence, such as constant routine and forced desynchrony studies, confirm the presence of an intrinsic rhythm in BP, independent of behavioral states, with peak levels occurring in the late evening circadian phase [5,28].

Peripheral clocks expressed in cardiovascular tissues, including the heart, vasculature, kidney, and autonomic nervous system, contribute to the temporal control of cardiovascular functions [26]. These clocks regulate genes involved in vascular reactivity, ion channel expression, and hormonal signaling. Disruption of peripheral clock gene expression, especially BMAL1, has been shown to impair normal circadian variation in vascular resistance, reduce baroreflex sensitivity, and promote pathological BP profiles [29,30]. Importantly, a loss of the nocturnal dipping pattern or the emergence of a “reverse dipping” phenotype is strongly associated with increased risk of cardiovascular events, target organ damage, and mortality [29,31].

### 2.3. Endothelial Function and Vascular Tone

Blood vessels are under circadian control at both the structural and functional levels. Vascular smooth muscle cells (VSMCs) and endothelial cells express core clock genes that regulate rhythmic gene transcription involved in vasomotor tone, extracellular matrix remodeling, and cell cycle progression [32]. Experimental deletion of *BMAL1* in VSMCs in murine models results in blunted circadian variation in systolic and diastolic BP, as well as loss of pulse pressure rhythmicity, highlighting the importance of local clocks in maintaining vascular homeostasis [33].

Endothelial-derived factors such as nitric oxide (NO) and endothelin-1 (ET-1) exhibit pronounced circadian patterns, contributing to time-of-day-dependent fluctuations in vascular tone. NO levels are typically lowest during the early morning hours, coinciding with the peak incidence of cardiovascular events, and highest in the late afternoon or evening [34]. Conversely, ET-1, a potent vasoconstrictor, follows a rhythmic secretion profile that aligns with sympathetic activity and modulates diurnal BP variability [35]. Circadian variations in peripheral blood flow, vascular resistance, and endothelial function underscore the role of the internal clock in optimizing cardiovascular efficiency and minimizing hemodynamic stress throughout the 24 h cycle [22].

### 2.4. Heart Rate and Autonomic Nervous System

HR follows a well-characterized circadian pattern, typically exhibiting lower values during nocturnal rest and higher values during daytime wakefulness [36]. This rhythm is governed not only by behavioral factors but also by endogenous circadian mechanisms that modulate the autonomic nervous system (ANS) [37]. Sympathetic activity tends to peak in the morning, contributing to the post-awakening surge in BP and HR. Meanwhile, parasympathetic (vagal) tone predominates during nighttime sleep, supporting cardiovascular recovery and energy conservation [38,39].

The circadian regulation of HR and HR variability (HRV) is mediated in part by oscillations in the expression of adrenergic receptors, ion channels, and autonomic regulatory genes, many of which are under the control of *CLOCK* and *BMAL1* [40]. In experimental models, disrupting these clock genes in cardiac tissue alters chronotropic responsiveness, reduces vagal modulation, and predisposes to arrhythmogenicity [41]. Interestingly, endogenous rhythms in circulating catecholamines, particularly norepinephrine and epinephrine, reach peak concentrations during midday. At the same time, vagal activity shows its highest levels in the early morning, revealing a phase difference that may have functional implications for cardiovascular risk timing [42].

## 3. Disruptions of Circadian Rhythms and Cardiovascular Disease Risk

### 3.1. Shift Work and Sleep Disorders

Modern societal demands have led to a growing proportion of the workforce, estimated at 15–27% in industrialized countries, engaging in nonstandard work schedules, including night shifts, rotating shifts, and extended-hour shifts [43]. Such shift work leads to chronic circadian misalignment, whereby behavioral cycles (e.g., sleep, activity, and feeding) are desynchronized from the endogenous circadian system. A large body of epidemiological evidence has demonstrated a significant association between shift work and increased risk of cardiovascular diseases (CVDs), including coronary heart disease (CHD) and stroke [44,45,46,47,48]. Shift work has been increasingly associated with adverse cardiovascular and metabolic health outcomes, as well as adverse psychological effects. Experimental and epidemiological studies highlight potential links between circadian disruption and increased risk of atherosclerosis, endothelial dysfunction, and impaired glucose metabolism. Moreover, prospective and mechanistic studies support the role of shift work as a contributor to long-term cardiovascular vulnerability and mental health burden [45,46,49,50].

The pathophysiological mechanisms linking shift work to CVD are multifactorial and involve complex interactions between sleep disruption, autonomic imbalance, metabolic dysregulation, and chronic low-grade inflammation [51,52,53]. Repeated alterations of the sleep–wake cycle impair sleep quality and duration, leading to increased sympathetic nervous system activity, elevated night-time BP, and reduced HRV, a marker of impaired parasympathetic tone [54]. Chronic misalignment between behavioral and circadian rhythms also disrupts glucose and lipid metabolism, promoting insulin resistance, hyperglycemia, and dyslipidemia [55]. Elevated levels of circulating inflammatory markers, such as C-reactive protein (CRP) and interleukin-6 (IL-6), have been observed in shift workers, further implicating inflammation as a mediator of vascular dysfunction [56]. Together, these alterations contribute to the development of atherosclerosis, hypertension, and other CVD risk factors.

Numerous longitudinal and meta-analytic studies demonstrate a dose–response relationship between the duration of shift work and cardiovascular risk. For example, each additional five years of shift work exposure has been associated with approximately a 5–7% increased risk of CVD overall, and a 4% increase in ischemic stroke risk in rotating night shift nurses. Moreover, individuals with long-term exposure (>15 years) show significantly higher incidences of both CHD and ischemic stroke [57,58,59]. This effect appears cumulative and progressive, particularly among individuals with rotating night shifts and irregular schedules. Notably, several large-scale cohort studies have shown that the association between shift work and CVD persists after adjustment for conventional risk factors such as hypertension, dyslipidemia, and type 2 diabetes [44,47,48]. These findings support the hypothesis that circadian misalignment, independently of other comorbidities, is causal in promoting CVD.

Given that a substantial proportion of the global workforce is regularly engaged in shift work, the public health implications of circadian disruption are profound. The increased cardiovascular risk observed in shift workers is not confined to clinical endpoints. Still, it is also reflected in a higher prevalence of intermediate risk factors, including obesity, metabolic syndrome, and elevated BP [15,45]. Importantly, the modifiability of work schedules, sleep hygiene, and behavioral timing offers potential avenues for intervention. Preventive strategies, such as optimizing shift rotations, limiting extended night shifts, and incorporating circadian-aware policies in occupational settings, may help mitigate the health burden associated with shift work [60]. Recognizing circadian misalignment as a distinct and targetable risk factor is essential for improving cardiovascular outcomes in this vulnerable population.

### 3.2. Lifestyle and Environmental Factors

Beyond occupational influences, a wide range of lifestyle and environmental factors have emerged as potent disruptors of the circadian system. Among them, ALAN, including exposure to dim light at night (dLAN) and blue-enriched wavelengths from electronic devices, has gained increasing attention due to its pervasiveness in modern society [61,62]. ALAN acts directly on the retinohypothalamic tract, suppressing melatonin synthesis via stimulation of the SCN and thereby altering circadian phase and amplitude [63]. Experimental and epidemiological studies have shown that nighttime light exposure reduces the normal day–night variability of BP and HR, largely by increasing nocturnal sympathetic activity and impairing parasympathetic recovery [28,64].

In both animal models and humans, ALAN exposure has been associated with higher resting BP, increased HR, and attenuated dipping patterns, which are all established risk factors for cardiovascular morbidity [65]. These physiological effects appear to be independent of sleep duration or quality, suggesting a direct circadian mechanism involving the ANS. Importantly, even low-intensity or short-duration light exposure, such as light from bedside screens or urban light pollution, can significantly impair circadian rhythmicity, emphasizing the need to consider ALAN as a modifiable cardiovascular risk factor [65,66].

In addition to light, feeding time is a critical external cue (zeitgeber) for synchronizing peripheral clocks in metabolic tissues, including the liver, pancreas, adipose tissue, and vasculature [25]. While the central circadian pacemaker in the SCN is primarily entrained by light, peripheral oscillators are highly responsive to meal timing and caloric intake. Disruption of this coordination, such as eating during the biological rest phase, can lead to desynchronization between central and peripheral clocks, a phenomenon linked to impaired glucose tolerance, altered lipid metabolism, and increased cardiometabolic risk [67].

Experimental studies in rodents have shown that feeding during the rest phase can shift peripheral clock gene expression, uncouple it from the SCN, and induce metabolic derangements even without changes in diet composition or caloric load [68]. In humans, late-night eating has been associated with higher BP, elevated postprandial glucose levels, and increased incidence of obesity and coronary artery disease (CAD). Conversely, time-restricted eating (TRE), limiting food intake to the active (light) phase of the circadian cycle, has demonstrated beneficial effects on body mass, BP, insulin sensitivity, and lipid profiles, even in the absence of caloric restriction [69,70].

Psychosocial stress, a ubiquitous feature of modern life, is another potent modulator of circadian rhythmicity and cardiovascular function. Acute and chronic stressors activate the hypothalamic–pituitary–adrenal axis (HPA), leading to rhythmic secretion of glucocorticoids such as cortisol in humans (or corticosterone in rodents), which themselves exhibit strong circadian variation, typically peaking in the early morning [71]. Disruption of circadian organization, for example, through shift work, ALAN, or jet lag, can disturb this glucocorticoid rhythm, leading to sustained hypercortisolemia, impaired feedback regulation, and elevated allostatic load, a physiological state associated with increased cardiovascular risk [72,73,74].

Moreover, stress interacts dynamically with circadian phase: identical stressors may produce markedly different cardiovascular responses depending on the time of day. Studies have shown that sympathetic activation and parasympathetic withdrawal are more pronounced in the early biological morning, a time when HRV is lowest and cardiovascular vulnerability is heightened [75]. Individuals experiencing high levels of occupational or emotional stress, especially shift workers, demonstrate blunted vagal tone, elevated baseline HR, and increased inflammatory cytokine levels, which collectively promote vascular dysfunction and atherogenesis [76].

The cumulative effects of ALAN exposure, mistimed feeding, and chronic stress converge on a state of circadian disruption, often referred to as circadian disruption. This condition is characterized by a persistent misalignment between endogenous circadian rhythms and environmental or behavioral timing cues [77]. Circadian disruption affects a wide array of biological systems, including the ANS, metabolic pathways, and vascular function, thereby facilitating the development of cardiometabolic and cardiovascular disorders [78]. This disruption plays a pivotal role in the pathogenesis of hypertension, metabolic syndrome, and obesity, forming a mechanistic link between lifestyle patterns and long-term cardiovascular risk.

### 3.3. Circadian Disruption and Cardiometabolic Syndromes

As it was mentioned above, circadian disruption refers to a sustained disturbance of a natural internal 24 h rhythm, often resulting from factors such as shift work, irregular sleep–wake schedules, nighttime light exposure, and mistimed food intake [79]. This disruption compromises the temporal coordination of biological processes, leading to a breakdown in synchronization between the central clock in the SCN and peripheral clocks in key metabolic tissues such as the liver, pancreas, adipose tissue, and vasculature [6]. Over time, this internal desynchrony contributes to metabolic inflexibility, promoting pathological states such as insulin resistance, dyslipidemia, visceral adiposity, and low-grade inflammation, which are hallmarks of cardiometabolic syndrome [79].

Epidemiological studies consistently show that individuals exposed to chronodisruptive conditions, particularly shift workers, late chronotypes, or those with irregular eating and sleeping habits, have a significantly increased risk of developing obesity, hypertension, type 2 diabetes, and metabolic syndrome [80,81]. These risks persist even when controlling for traditional lifestyle factors, suggesting that circadian misalignment itself is an independent contributor to cardiometabolic pathology [82].

The relationship between obesity and circadian disruption is bidirectional: while misaligned circadian rhythms can promote weight gain and metabolic dysfunction, obesity itself further impairs circadian regulation at both systemic and cellular levels [83]. Adipose tissue possesses autonomous circadian clocks that regulate the transcription of genes involved in lipogenesis, adipokine secretion, and insulin sensitivity [84]. Disruption of clock gene expression, particularly of *BMAL1* and *CLOCK*, has been associated with increased adiposity, impaired glucose tolerance, and altered lipid profiles in both animal and human studies [19].

Experimental models have demonstrated that mice subjected to chronic light–dark cycle perturbations or fed during the rest phase develop significant weight gain, hepatic steatosis, and insulin resistance, even under isocaloric conditions. Similarly, clinical data show that individuals with late chronotypes, irregular sleep patterns, or nighttime eating habits exhibit a higher prevalence of visceral obesity and metabolic syndrome [85,86]. These findings highlight the role of circadian disruption not merely as a consequence, but as an active driver of metabolic disease.

In light of the strong link between circadian disruption and metabolic dysfunction, TRE has emerged as a promising non-pharmacological intervention to restore circadian alignment and improve cardiometabolic outcomes. TRE involves confining daily caloric intake to a limited window, typically 8–12 h, during the active (daylight) phase, without necessarily altering the quantity or quality of food consumed [19,87]. This feeding pattern reinforces peripheral clock gene oscillations and re-establishes synchrony between the central and peripheral circadian systems.

Both animal and human studies have shown that TRE can lead to reductions in body mass, waist circumference, BP, and atherogenic lipid levels, as well as improvements in insulin sensitivity and inflammatory markers, even in the absence of caloric restriction or increased physical activity [88,89]. These benefits are thought to arise from the reorganization of metabolic timing, improved mitochondrial function, and enhanced rhythmic gene expression [88]. As such, TRE represents a simple yet effective chronotherapeutic strategy to mitigate the adverse metabolic effects of circadian misalignment.

Circadian disruption plays a central role in the pathogenesis and progression of cardiometabolic disorders, acting through mechanisms that include endocrine imbalance, autonomic dysregulation, oxidative stress, and inflammatory activation [90]. The recognition that “when” physiological processes occur is as important as “what” occurs has led to a paradigm shift in cardiometabolic research and clinical practice. Understanding the biological consequences of sleep disturbances, mistimed feeding, and light-at-night exposure is essential for identifying at-risk individuals and designing targeted lifestyle interventions.

Realignment of circadian rhythms through strategies such as TRE, improved sleep hygiene, and light environment optimization offers promising avenues for preventing and managing conditions such as hypertension, obesity, and metabolic syndrome. Integrating chronobiological principles into cardiovascular prevention frameworks could enhance therapeutic efficacy and reduce long-term disease burden.

## 4. The Role of Melatonin in Cardiovascular Health

### 4.1. Melatonin Biosynthesis and Secretion

Melatonin (N-acetyl-5-methoxytryptamine) is an evolutionarily conserved indoleamine derived from the essential amino acid tryptophan [91]. Although this molecule is synthesized across diverse taxa, including plants, bacteria, and fungi, it plays a particularly vital role in vertebrates as a hormonal signal of darkness [91]. In mammals, melatonin secretion displays a robust circadian rhythm, with low daytime levels and peak nocturnal concentrations that convey information about the light–dark cycle to peripheral tissues. By acting as a systemic cue of circadian phase, melatonin helps coordinate internal biological rhythms with external environmental conditions [92].

In mammals, the pineal gland is the principal source of circulating melatonin, and its activity is tightly regulated by the central circadian pacemaker located in the SCN of the hypothalamus [2]. The SCN receives photic input from intrinsically photosensitive retinal ganglion cells via the retinohypothalamic tract and transmits this information to the pineal gland through a multisynaptic sympathetic pathway involving the paraventricular nucleus, intermediolateral cell column, and superior cervical ganglion [93]. During darkness, sympathetic stimulation of the pineal gland triggers norepinephrine release, which activates arylalkylamine N-acetyltransferase (AANAT), the rate-limiting enzyme in melatonin synthesis, resulting in a nocturnal surge in melatonin levels [94].

Exposure to light at night, particularly short-wavelength blue light (450 nm), suppresses melatonin synthesis by inhibiting this neural pathway, leading to attenuated nocturnal secretion and phase shifts in circadian rhythms [93]. Even low-intensity artificial light can significantly disrupt melatonin production, underscoring its sensitivity to environmental illumination [95].

Melatonin production exhibits a clear age-related decline, with peak nocturnal levels diminishing progressively over the human lifespan. In older individuals, this reduction is attributed to both functional deterioration of the pineal gland and structural changes within the SCN, including neuronal loss and reduced responsiveness to photic input [96]. As melatonin plays a central role in synchronizing circadian rhythms, its age-related decline may contribute to the flattening of hormonal and physiological rhythms, fragmentation of sleep, and increased susceptibility to cardiometabolic dysfunction in older adults.

**Figure 1:**
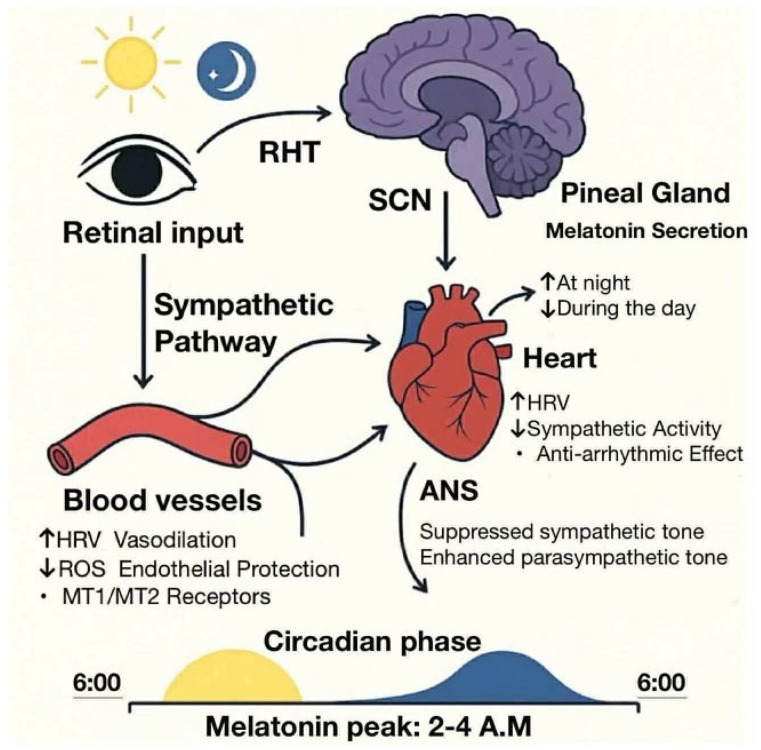
The suprachiasmatic nucleus (SCN)–melatonin axis and its cardiovascular effects. Light input is received by the retina and transmitted to the SCN via the retinohypothalamic tract. As the master circadian pacemaker, the SCN regulates melatonin secretion from the pineal gland, increasing at night and decreasing during the day. Melatonin modulates the autonomic nervous system (ANS), suppressing sympathetic tone and enhancing parasympathetic activity. It also acts directly on the heart and vasculature, improving heart rate variability (HRV), reducing sympathetic drive, exerting anti-arrhythmic effects, promoting vasodilation, protecting the endothelium, and reducing the levels of reactive oxygen species (ROS), partly via melatonin receptor subtypes 1 and 2 (MT1 and MT2). Melatonin levels peak between 2:00 and 4:00 A.M., aligning with endogenous circadian rhythms. ↑—increased; ↓—decreased.

Lifestyle and environmental factors also exert a strong modulatory influence on melatonin secretion. Nocturnal exposure to artificial light, especially blue-enriched light from electronic devices and light-emitting diode (LED) sources, suppresses endogenous melatonin release by interfering with the SCN-mediated sympathetic signaling to the pineal gland [97]. Moreover, irregular sleep–wake schedules, shift work, and insufficient exposure to natural daylight exacerbate circadian misalignment, leading to blunted melatonin rhythms and promoting circadian disruption [24]. These alterations are increasingly recognized as contributing factors in the pathogenesis of cardiovascular and metabolic diseases. An overview of the SCN–melatonin pathway and its influence on cardiovascular regulation is presented in Figure 1.

### 4.2. Cardioprotective Properties of Melatonin

The most extensively studied cardioprotective mechanisms of melatonin include its potent antioxidant capacity, which it exerts through both direct and indirect pathways. Melatonin is capable of directly scavenging highly reactive ROS and RNS, including hydroxyl radicals, peroxynitrite, and singlet oxygen [98]. In addition, melatonin stimulates the expression and activity of endogenous antioxidant enzymes, such as SODs, GPxs, and catalases, enhancing the cellular defense system against oxidative stress [99].

Melatonin’s antioxidant cascade is its distinctive feature. The metabolic byproducts generated during melatonin scavenging retain radical-neutralizing properties, thereby prolonging and amplifying the antioxidant effect. Moreover, those byproducts have stronger antioxidant properties than melatonin. This multi-layered defense system is particularly relevant in the cardiovascular system, where oxidative stress contributes to endothelial dysfunction, lipid peroxidation, vascular inflammation, and atherogenesis [99].

In addition to its antioxidant properties, melatonin exerts significant anti-inflammatory effects, which contribute to its cardioprotective profile. One of the primary mechanisms involves inhibition of the nuclear factor kappa B (NF-κB) signaling pathway, a central regulator of pro-inflammatory gene expression [100]. By suppressing NF-κB activation, melatonin reduces the transcription of pro-inflammatory cytokines such as tumor necrosis factor-alpha (TNF-α), IL-6, and interleukin-1beta (IL-1β), thereby mitigating vascular inflammation and preventing the progression of atherogenic processes [101].

Interestingly, melatonin modulates immune system function by influencing the activity of macrophages, T lymphocytes, and other immune effector cells. This immunomodulatory effect helps maintain immune homeostasis, promoting adequate host defense while limiting chronic low-grade inflammation, a key driver of endothelial dysfunction, plaque instability, and cardiovascular events [12].

Melatonin also plays a critical role in the regulation of BP and vascular tone, acting through both central nervous system mechanisms and direct vascular effects. Centrally, melatonin modulates the ANS activity, notably by reducing sympathetic outflow, which leads to decreased vascular resistance and lower resting BP [102]. Peripherally, melatonin binds to melatonin receptor subtypes 1 (MT1) and 2 (MT2) expressed on VSMCs and endothelial cells, promoting vasodilation through increased NO bioavailability and reduced ROS generation [103].

Both MT1 and MT2 receptors are expressed in cardiovascular tissues, including endothelial cells, vascular smooth muscle cells, and cardiomyocytes [7]. MT1 activation has been associated with vasoconstrictive effects in certain vascular beds, mediated through Gi/o-protein-coupled inhibition of adenylate cyclase, reduced cAMP production, and increased intracellular Ca^2+^ in smooth muscle cells. In contrast [104], MT2 activation is more frequently linked to vasodilatory responses, in part by stimulating endothelial nitric oxide synthase activity, enhancing NO bioavailability, and promoting opening of large-conductance Ca^2+^-activated K^+^ channels [7,105]. Both receptors also modulate oxidative stress and autonomic balance, contributing to the circadian regulation of vascular tone and blood pressure. These complementary and sometimes opposing effects underscore the importance of receptor-specific pathways in mediating melatonin’s cardiovascular actions [7].

These vascular actions contribute to the preservation of endothelial function, a key determinant of cardiovascular health. By counteracting oxidative stress and inhibiting inflammatory signaling, melatonin protects the endothelial glycocalyx, supports arterial elasticity, and limits early vascular remodeling. The cumulative effects of these actions include a reduction in arterial stiffness, improved baroreflex sensitivity, and stabilization of circadian BP rhythms, particularly beneficial in hypertensive patients with a blunted nocturnal dip. The key molecular targets and physiological mechanisms underlying the cardioprotective actions of melatonin are summarized in Table 1.

### 4.3. Melatonin and Chronotherapy for CVD

Chronotherapy refers to the strategic timing of treatment administration in alignment with the body’s biological rhythms to optimize therapeutic efficacy and minimize adverse effects. In cardiovascular medicine, this concept is particularly relevant, as the incidence of adverse events such as myocardial infarction, stroke, and sudden cardiac death exhibits marked circadian patterns, often peaking in the early morning hours [106]. Melatonin, as a central modulator of circadian timing and vascular homeostasis, has emerged as a promising candidate for circadian-targeted intervention in disorders such as hypertension and atherosclerosis [7].

By influencing multiple pathophysiological pathways, including oxidative stress, endothelial dysfunction, autonomic imbalance, and inflammatory activation, melatonin provides a multifaceted therapeutic profile. Importantly, the timing of melatonin administration determines its physiological impact, highlighting the relevance of chronobiological principles in its clinical use [114].

Clinical studies have demonstrated that evening supplementation with melatonin, typically administered 1–2 h before bedtime, can significantly reduce nocturnal BP, particularly in individuals with a non-dipping profile, those who fail to exhibit the normal nighttime decline in BP. This subgroup is at increased risk for target organ damage and cardiovascular events, making it a key target for chronotherapeutic interventions [115,116]. As it was presented above, melatonin can exert its antihypertensive effects through several mechanisms, including attenuation of sympathetic tone, enhancement of parasympathetic activity, and improvement of endothelial function [102]. Notably, studies comparing daytime vs. nighttime dosing have shown that evening administration results in greater reductions in systolic and diastolic BP, improved sleep quality, and better alignment with endogenous circadian rhythms [117].

Beyond its antihypertensive actions, melatonin has demonstrated atheroprotective effects in preclinical studies. It inhibits the oxidation of low-density lipoprotein (LDL) cholesterol, a key initiating step in atherogenesis, and reduces the formation of foam cells by preventing lipid accumulation in macrophages [109]. Additionally, melatonin suppresses the expression of adhesion molecules and pro-inflammatory cytokines in the vascular endothelium, thereby limiting monocyte infiltration and plaque development [110]. Animal studies using atherosclerosis-prone models have shown that melatonin supplementation reduces the size and number of atherosclerotic lesions, stabilizes plaque structure, and decreases markers of vascular inflammation [110]. These effects are largely attributed to the inhibition of NF-κB-dependent signaling, reduction in oxidative stress, and restoration of endothelial integrity [118]. Although human data remain limited, these findings highlight the potential of melatonin as an adjunctive agent in atherosclerosis prevention and management.

The timing of melatonin administration plays a pivotal role in determining its therapeutic efficacy, particularly in cardiovascular applications. Administering melatonin in synchrony with the endogenous circadian rhythm, typically during the biological night, enhances its ability to regulate BP, reduce oxidative stress, and restore endothelial function [111]. In contrast, daytime supplementation appears less effective and may even disrupt circadian homeostasis. These observations support the concept of melatonin-based chronotherapy, in which treatment is aligned with diurnal variations in cardiovascular risk [112]. By targeting periods of increased vulnerability, such as the early morning surge in sympathetic activity, chronotherapeutic use of melatonin holds promise for reducing cardiovascular events, particularly in high-risk populations. While further clinical trials are needed to establish optimal dosing regimens and long-term outcomes, current evidence suggests that melatonin may serve as a safe, low-cost, and physiologically compatible adjunct in the prevention and management of hypertension and atherosclerosis, especially when administered with circadian precision.

Although several clinical trials and meta-analyses have reported reductions in nocturnal blood pressure and improvements in sleep quality following evening melatonin supplementation, the overall evidence remains mixed. The meta-analysis by Grossman et al. [116] demonstrated that controlled-release melatonin (2–5 mg for 7–90 days) significantly lowered nocturnal systolic and diastolic blood pressure in hypertensive patients, whereas immediate-release formulations generally showed no effect on 24 h ambulatory blood pressure. In contrast, some studies have not observed cardiovascular benefits, for example, a six-week randomized trial in older women with insomnia found no improvement in arterial stiffness or mitochondrial DNA markers despite within-group reductions in systolic blood pressure [119], and a high-dose (24 mg) crossover trial in African-American patients with hypertension reported no change in nocturnal blood pressure [120]. Mild and transient adverse effects, such as daytime somnolence, vivid dreams, or headache, have also been noted [116]. Variability in findings may be explained by differences in formulation, dosage, patient phenotyping, treatment duration, baseline dipping status, and outcome measurement methods.

Typical dosing regimens in cardiovascular studies involve evening administration of 2–5 mg melatonin, most often in controlled-release formulations, taken 1–2 h before habitual bedtime for intervention periods ranging from 2 weeks to 12 months [115,121]. Immediate-release preparations have shown less consistent effects on nocturnal blood pressure and other cardiovascular outcomes [121]. Short-term supplementation appears to be well tolerated, with mild adverse events such as daytime somnolence, vivid dreams, and headache reported in a minority of participants, and no serious cardiovascular complications observed in the available literature [122]. However, data on long-term safety in cardiovascular populations remain limited, and potential drug interactions—particularly with sedative agents or anticoagulant/antiplatelet medications—should be considered [122]. At present, the optimal dose, formulation, and duration of melatonin therapy for cardiovascular indications remain to be determined and require confirmation in well-powered, rigorously phenotyped, randomized controlled trials.

Taken together, the current body of evidence on melatonin chronotherapy for cardiovascular disease is constrained by important methodological limitations. Preclinical data are extensive and consistent in demonstrating antihypertensive and vasoprotective effects via multiple mechanisms [7], but translation into clinical benefit relies on relatively few human studies. Most clinical trials to date have been small (*n* ≈ 16–47), short-term (≤4 weeks), and often single-center, with only a minority at low risk of bias [115]. Meta-analyses pooling these trials indicate that the nocturnal BP-lowering effect is observed primarily with controlled-release melatonin, whereas immediate-release preparations show no benefit [115,116]. The certainty of evidence has been rated low to very low owing to imprecision and quality limitations [115]. Variability in study design, dosing regimens, patient selection, and outcome measures further limits comparability and generalizability [115]. These factors underscore the need for adequately powered, long-duration randomized controlled trials, particularly in well-characterized patient subgroups, to confirm efficacy, optimize treatment parameters, and determine long-term cardiovascular outcomes [115,116].

## 5. Clinical and Public Health Implications

Growing evidence supports the inclusion of circadian disruption as an independent and modifiable risk factor in the clinical assessment of CVD. Traditionally, risk stratification has focused on static variables such as BP, cholesterol levels, and smoking status [123]. However, temporal patterns of physiological function, including non-dipping BP profiles, altered HRV, and disrupted sleep–wake cycles, have been increasingly associated with adverse cardiovascular outcomes [28]. Patients with non-dipping or reverse-dipping BP exhibit higher rates of left ventricular hypertrophy, microalbuminuria, and stroke, underscoring the prognostic value of circadian patterns in cardiovascular medicine [124].

Incorporating circadian parameters into clinical diagnostics may improve the accuracy of cardiovascular risk assessment and guide timing-specific interventions. For example, 24 h ambulatory BP monitoring (ABPM) can identify non-dipping patterns and nocturnal hypertension, which are frequently missed in office measurements yet strongly predictive of cardiovascular events. Similarly, HRV, particularly its circadian rhythm, is a sensitive marker of autonomic dysfunction and may provide early warning of increased sympathetic tone or diminished vagal activity [107,108].

Therapeutically, timing the administration of cardiovascular medications to align with circadian patterns, an approach known as chronotherapy, has shown promising results [113]. Evening dosing of antihypertensive agents, such as angiotensin converting enzyme (ACE) inhibitors or angiotensin receptor blockers (ARBs), may better restore nocturnal BP dipping and improve endothelial function [125]. Furthermore, the use of melatonin or melatonergic agonists in selected patient groups, such as individuals with resistant hypertension or circadian sleep disorders, represents a low-risk, potentially high-benefit adjunctive strategy [12].

Personalized cardiovascular care may benefit from the integration of individual circadian profiles, such as chronotype, sleep–wake regularity, and exposure to environmental light. Patients with late chronotypes, irregular sleep schedules, or shift work history may require tailored approaches that take into account their unique circadian vulnerabilities [126]. Despite promising findings, circadian-based interventions are not yet widely adopted in routine cardiology, due in part to a lack of large-scale randomized trials and standardized clinical guidelines.

To bridge this gap, future research should focus on defining biomarkers of circadian disruption, validating time-of-day-dependent treatment responses, and identifying patient subgroups most likely to benefit from chronotherapy. Incorporating circadian metrics into clinical decision-making tools has the potential to enhance risk prediction, optimize treatment timing, and ultimately reduce the burden of CVD.

Circadian disruption is no longer a niche biomedical concern, but a widespread population-level issue fueled by industrialization, artificial lighting, digital screen exposure, and 24 h work schedules. Epidemiological data indicate that shift work is a common feature of modern labor markets in developed countries, while exposure to ALAN continues to increase due to expanding urban environments and the pervasive use of screen-based technologies [43]. These environmental factors, combined with behavioral patterns such as late-night eating, shortened sleep duration, and irregular social schedules, contribute to a global rise in chronodisruption-associated cardiometabolic risk.

Given its cumulative and often subclinical progression, circadian misalignment is difficult to detect and under-recognized in public health planning. However, its links to major non-communicable diseases, including hypertension, diabetes, stroke, and CAD, warrant targeted public health interventions [127].

Public health strategies aimed at mitigating circadian disruption should target modifiable environmental and behavioral risk factors. Key interventions include limiting exposure to ALAN, especially blue-light-emitting screens, by promoting screen-free periods before bedtime, encouraging the use of blue-light filters, and improving urban lighting policies to reduce nighttime light pollution [128]. At the workplace level, minimizing long-term night shift rotations, enabling predictable scheduling, and facilitating natural daylight exposure during waking hours can help preserve circadian alignment among shift workers [129].

In addition, public health messaging should emphasize the importance of sleep regularity, consistent meal timing, and time-appropriate physical activity as core elements of cardiovascular health promotion. Educational programs targeting both the general population and healthcare professionals are needed to raise awareness of “circadian hygiene”, analogous to sleep hygiene, as a component of lifestyle medicine [130]. Integrating chronobiological insights into national prevention guidelines may enhance efforts to curb the rising prevalence of cardiometabolic diseases. Key circadian disruptors, their biological impact, cardiovascular risk, and potential interventions are summarized in Table 2.

Addressing circadian disruption at the population level will require multisectoral collaboration, bridging clinical medicine, occupational health, urban planning, and public policy. Designing built environments that prioritize access to natural light, regulating nighttime work conditions, and supporting family- and circadian-friendly workplace policies are essential steps toward long-term cardiovascular prevention. Likewise, incorporating chronobiological principles into public health guidelines, occupational health assessments, and environmental regulations can facilitate the emergence of circadian-conscious infrastructures [131].

Just as tobacco control and dietary reform have reshaped public health norms over the past decades, protecting biological time may represent the next frontier in chronic disease prevention. Recognizing circadian health as a public good and designing systems that protect it holds significant potential for reducing the global burden of cardiovascular and metabolic diseases.

Preliminary clinical protocols in cardiovascular chronotherapy have also explored the evening administration of selected antihypertensive agents, such as ACE inhibitors, angiotensin receptor blockers, and calcium channel blockers, to better align pharmacological effects with circadian BP variability. Some studies have reported enhanced nocturnal BP reduction and improved dipping status with such regimens [132,133], while others found no significant difference compared to morning dosing [134]. Given this heterogeneity and the potential for patient-specific responses, current recommendations emphasize individualized timing based on ambulatory BP monitoring and clinical context. Within this framework, controlled-release melatonin may serve as an adjunctive option in patients with blunted nocturnal BP decline or persistent nocturnal hypertension [115], although formal guideline endorsement awaits further high-quality evidence.

**Figure 2:**
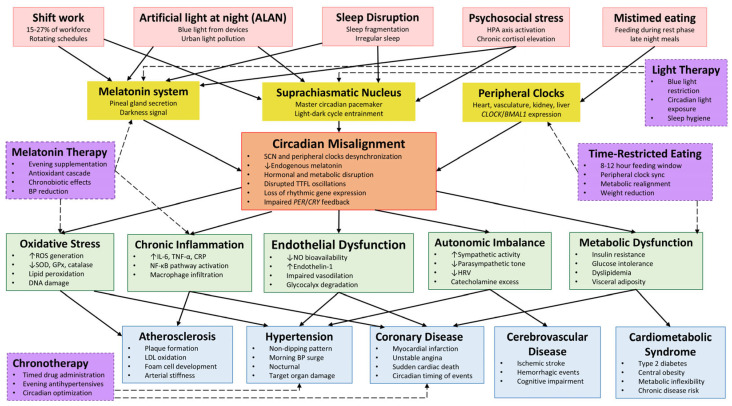
Graphical representation of the cascade linking circadian disruption to cardiovascular disease. Environmental and behavioral disruptors initiate circadian misalignment, which in turn promotes oxidative stress, inflammation, autonomic imbalance, and metabolic dysfunction. These pathophysiological mechanisms contribute to hypertension, atherosclerosis, and other cardiovascular outcomes. Points of potential intervention—such as chronotherapy, time-restricted eating, and melatonin supplementation—are highlighted with dashed arrows. Abbreviations used: ALAN—artificial light at night; ANS—the autonomic nervous system; BP—blood pressure; *CLOCK*—circadian locomotor output cycles kaput; CRP—C-reactive protein; HRV—heart rate variability; IL-6—interleukin-6; MT_1_/MT_2_—melatonin receptor subtypes 1/2; NF-κB—nuclear factor kappa B; NO—nitric oxide; *PER*/*CRY*—period/cryptochrome (clock gene families); ROS—reactive oxygen species; SCN—the suprachiasmatic nucleus; SOD—superoxide dismutase; TTFL—transcriptional–translational feedback loop.

A comprehensive overview of the pathophysiological cascade linking circadian disruption to cardiovascular outcomes is presented in Figure 2.

## 6. Limitations and Future Directions

Despite growing evidence linking circadian disruption to CVD, several knowledge gaps remain that limit the translation of these findings into effective interventions. While studies have implicated mechanisms such as oxidative stress, inflammation, and endothelial dysfunction, the precise molecular pathways through which circadian misalignment drives cardiovascular pathology are incompletely understood [135]. For example, the functional interactions between central and peripheral clocks, and their modulation by behavioral or environmental cues, are still being actively investigated. Moreover, the pleiotropic actions of melatonin in the cardiovascular system are difficult to disentangle from its circadian effects, complicating the design of targeted therapies [136].

Adding further complexity, circadian responses vary considerably between individuals, influenced by factors such as age, sex, genetic background, chronotype, and comorbid conditions. These interindividual differences pose challenges for the development of universal diagnostic criteria or therapeutic standards, underscoring the need for personalized approaches to circadian-based cardiovascular care [137].

The implementation of circadian-based strategies into routine cardiovascular care faces significant translational challenges. The lack of clinically practical tools to assess circadian phase or rhythm integrity is one of the major barriers. While laboratory-based measures, such as dim light melatonin onset (DLMO), core body temperature profiles, and 24 h hormonal sampling, are effective in research, they are often impractical or unavailable in clinical settings [138]. Additionally, widely used diagnostic procedures, such as BP measurements or lipid panels, are rarely standardized by time of day, which may obscure meaningful circadian variations [139].

Another obstacle lies in the limited awareness and training among healthcare professionals regarding the relevance of chronobiology in CVD. Current clinical guidelines do not yet incorporate time-of-day–dependent risk assessment or treatment planning, and few clinical trials have rigorously evaluated chronotherapeutic regimens for CVD. As a result, even well-established interventions, such as evening dosing of antihypertensives or melatonin supplementation, remain underutilized, despite promising evidence [140].

To advance the field of circadian cardiology, future research should focus on the development of cost-effective and widely implementable biomarkers that reflect circadian phase and rhythmic integrity, such as salivary melatonin, cortisol profiles, or wearable-derived HRV metrics. These tools should be simple, scalable, and compatible with routine care, enabling clinicians to objectively assess circadian alignment and tailor interventions accordingly [141,142]. In parallel, large-scale, randomized controlled trials are needed to evaluate the efficacy of chronotherapy, including time-of-day-specific drug administration and lifestyle interventions such as TRE or light exposure therapy.

Another important step will be the integration of circadian variables into clinical risk models and electronic health records, allowing for a more personalized and temporally informed approach to care. Public health initiatives and medical education programs should also be developed to promote awareness of biological rhythms as a determinant of cardiovascular risk [143]. Ultimately, aligning preventive and therapeutic strategies with the body’s internal timing mechanisms may offer a powerful yet underutilized pathway toward reducing the burden of CVD.

## 7. Conclusions

Circadian rhythms profoundly influence cardiovascular physiology by regulating key parameters such as blood pressure, heart rate, vascular tone, and metabolic function. Disruption of these rhythms, whether due to behavioral, occupational, or environmental factors, has emerged as an independent contributor to the development and progression of cardiovascular disease. Mounting evidence from experimental, clinical, and epidemiological studies underscores the role of circadian misalignment in promoting endothelial dysfunction, oxidative stress, inflammation, and metabolic dysregulation.

Melatonin, a neuroendocrine hormone and key chronobiotic signal, stands out as a promising agent with multifaceted cardioprotective effects. Its antioxidant, anti-inflammatory, and autonomic-modulating properties, along with its role in circadian synchronization, position melatonin as both a mechanistic link and a potential therapeutic tool in the context of cardiovascular chronomedicine.

To fully realize the clinical potential of circadian-based strategies, future efforts must focus on the development of accessible circadian biomarkers, time-sensitive diagnostic tools, and evidence-based chronotherapeutic protocols. Incorporating circadian biology into cardiovascular care frameworks and increasing awareness among clinicians and the public will be essential to optimizing prevention and treatment strategies in an increasingly 24 h society.

## Figures and Tables

**Table 1:** Molecular mechanisms and physiological effects underlying the cardioprotective actions of melatonin.

Mechanism of Action	Main Molecular Targets/Signaling Pathways	Physiological/Clinical Effect	Type of Evidence	References
Antioxidant effect	ROS, hydroxyl radicals, peroxynitrite, SOD, GPx, catalase	Reduction of oxidative stress, endothelial protection, prevention of oxidative vascular damage	Preclinical/Clinical	[98,99]
Anti-inflammatory action	NF-κB, pro-inflammatory cytokines (TNF-α, IL-6, IL-1β)	Inflammation reduction, atherosclerosis progression delay, plaque stabilization	Preclinical/Clinical	[100,101]
Immunomodulation	Immune cells (macrophages, T lymphocytes)	Maintenance of immune homeostasis, attenuation of low-grade chronic inflammation	Preclinical	[12]
Autonomic regulation	Sympathetic nervous system, central nervous system	Blood pressure reduction, vascular resistance decrease, sympathetic activity modulation	Preclinical/Clinical	[102]
Receptor-mediated vasodilation	MT1 and MT2 receptors, nitric oxide (NO), ROS	Vasodilation, improved blood flow, enhanced NO bioavailability	Preclinical	[103]
Endothelial protection	Anti-inflammatory and antioxidant signaling, endothelial glycocalyx	Improved vascular elasticity, reduced remodeling, enhanced baroreflex function	Preclinical/Clinical	[103]
Circadian resynchronization	Circadian blood pressure rhythm, clock gene activity	Restoration of nocturnal blood pressure dip, improved chronobiological regulation in hypertensive patients	Clinical	[106]
Regulation of circadian rhythm and HRV	*CLOCK*, *BMAL1*, adrenergic receptors, catecholamine rhythmicity, cardiac autonomic regulation	Stabilization of heart rate and blood pressure rhythms, improved HRV, reduced sympathetic overactivation risk	Preclinical/Clinical	[12,28,107,108]
Anti-atherosclerotic effect	LDL oxidation, foam cell formation, NF-κB pathway, adhesion molecules, circadian-timed administration	Inhibition of atherosclerosis progression, plaque stabilization, endothelial protection, enhanced effect with nighttime dosing	Preclinical/Clinical	[109,110,111,112]
Chronotherapeutic effect	Circadian timing, *CLOCK*/*BMAL1*,	Enhanced antihypertensive and endothelial effects when administered at night	Clinical	[111,112,113]

Abbreviations: *BMAL1*—brain and muscle aryl hydrocarbon receptor nuclear translocator (Arnt)-like protein 1; *CLOCK*—circadian locomotor output cycles kaput; GPx—glutathione peroxidase; HRV—heart rate variability; IL-1β—interleukin-1 beta; IL-6—interleukin-6; LDL—low-density lipoprotein; MT1/MT2—melatonin receptor subtypes 1/2; NF-κB—nuclear factor kappa B; NO—nitric oxide; ROS—reactive oxygen species; SCN—the suprachiasmatic nucleus; SOD—superoxide dismutase; TNF-α—tumor necrosis factor alpha.

**Table 2:** Circadian disruptors, their physiological consequences, cardiovascular risk implications, and evidence-based interventions.

Circadian Disruptor	Mechanism of Rhythm Disruption	Physiological Effects	CVD Risk Evidence (Literature-Based)	Evidence-Based Interventions	References
Shift Work	SCN-peripheral clock desynchronization; disrupted sleep-wake cycles; chronic behavioural misalignment	↑ sympathetic activity; ↓ HRV; ↑ nocturnal BP; ↑ IL-6, CRP; insulin resistance; Sleep fragmentation	5–7% ↑ CVD risk per 5 years exposure; 4% ↑ ischemic stroke risk; ↑ CHD in rotating night shifts; dose-response relationship with duration	Optimized shift rotations; limited extended night shifts; bright light during work; sleep hygiene protocols; melatonin administration timing	[12,44,47,48,57,58,59,60,115,129]
Artificial Light at Night (ALAN)	Retinohypothalamic tract stimulation; melatonin suppression via SCN inhibition; phase shifts in circadian timing	Loss of nocturnal BP dipping; ↑ resting BP and HR; ↓ parasympathetic recovery; Disrupted glucose tolerance; ↑ cortisol	Attenuated dipping patterns; ↑ cardiovascular morbidity independent of sleep quality; ↑ hypertension risk from bedroom light exposure	Blue light filters; screen-free periods before bedtime; dim red lighting; urban lighting policies; circadian lighting design	[61,64,65,66,93,97,128,131]
Mistimed Eating/Late-Night Feeding	Peripheral clock desynchronization; uncoupling from SCN; disrupted metabolic gene expression; feeding-induced phase shifts	↑ postprandial glucose; ↑ BP; metabolic inflexibility; ↑ visceral adiposity; altered lipid profiles; insulin resistance	↑ Obesity and CAD risk; metabolic syndrome development; ↑ CVD events in late chronotypes	Time-restricted eating (8–12 h window); consistent meal timing; avoiding eating 3 h before sleep; alignment of feeding with active phase	[67,68,69,70,85,86,87,88,89]
Chronic Psychosocial Stress	HPA axis activation; disrupted cortisol rhythm; glucocorticoid receptor dysregulation; SCN-stress system interactions	Sustained hypercortisolemia; ↑ allostatic load; ↓ HRV; ↑ inflammatory markers; Autonomic imbalance; impaired sleep	↑ CVD vulnerability; time-dependent stress responses; amplified morning cardiovascular events; ↑ atherosclerosis progression	Stress management techniques; cortisol rhythm restoration; circadian-timed stress reduction	[71,72,73,74,75,76,130]
Sleep Disruption/Irregular Sleep	Fragmented sleep architecture; reduced sleep efficiency; circadian phase instability; SCN input disruption	↑ sympathetic tone; ↓ parasympathetic activity; ↑ inflammatory cytokines; Glucose intolerance; ↑ BP variability	Strong predictor of CVD events; ↑ hypertension; ↑ stroke risk; metabolic dysfunction; accelerated atherosclerosis	Sleep hygiene optimization; consistent sleep-wake timing; sleep environment control; cognitive behavioral therapy for insomnia	[51,53,54,60,130]
Age-Related Circadian Decline	Pineal gland deterioration; SCN neuronal loss; ↓ photic responsiveness; blunted rhythm amplitude	Progressive melatonin decline; flattened hormonal rhythms; ↓ sleep quality; ↑ fragmentation; metabolic dysregulation	↑ CVD risk in elderly; accelerated vascular aging; ↑ hypertension prevalence; metabolic syndrome	Melatonin replacement therapy; bright light therapy; activity scheduling; chronotherapy protocols	[44,51,65,73,85,86,93,96,125,131]
Urban Light Pollution	Chronic low-level ALAN exposure; disrupted darkness signal; ecological circadian disruption	Suppressed melatonin synthesis; altered sleep patterns; metabolic rhythm disruption; immune dysfunction	Population-level CVD risk increase; environmental circadian disruption; metabolic health impacts	Urban lighting regulations; shielded lighting; reduced brightness standards; circadian-conscious city planning	[63,128,131]
Electronic Device Usage	Blue light emission (450 nm); evening circadian phase delays; melatonin suppression; sleep onset disruption	Delayed sleep phase; ↓ sleep quality; ↑ evening alertness; Disrupted morning cortisol rhythm	Association with metabolic dysfunction; ↑ obesity risk; cardiovascular risk markers	Screen time limits; blue light blocking; device-free bedrooms; evening usage restrictions	[93,97,128,131]

Abbreviations: ALAN—artificial light at night; BP—blood pressure; CAD—coronary artery disease; CHD—coronary heart disease; CRP—C-reactive protein; CVD—cardiovascular disease; HRV—heart rate variability; HPA—hypothalamic–pituitary–adrenal axis; IL-6—interleukin-6; SCN—suprachiasmatic nucleus; ↑—increased; ↓—decreased.

## Data Availability

Data are contained within the article.

## Abbreviations

The following abbreviations are used in this manuscript:

AANAT	arylalkylamine N-acetyltransferase
ABPM	ambulatory blood pressure monitoring
ACE	angiotensin converting enzyme
ALAN	artificial light at night
ANS	the autonomic nervous system
ARB	angiotensin receptor blocker
Arnt	aryl hydrocarbon receptor nuclear translocator
BMAL1	brain and muscle Arnt-like protein 1
BP	blood pressure
CAD	coronary artery disease
CHD	coronary heart disease
CLOCK	circadian locomotor output cycles kaput
CRP	C-reactive protein
CRY	cryptochrome
CVD	cardiovascular disease
dLAN	dim light at night
DLMO	dim light melatonin onset
E-box	enhancer box
ET-1	endothelin-1
GPx	glutathione peroxidase
HPA	the hypothalamic–pituitary–adrenal axis
HR	heart rate
HRV	heart rate variability
IL-1β	interleukin-1beta
IL-6	interleukin-6
LDL	low-density lipoprotein
LED	light-emitting diode
MT1	melatonin receptor subtype 1
MT2	melatonin receptor subtype 2
NF-κB	nuclear factor kappa B
NO	nitric oxide
NR1D1	nuclear receptor subfamily 1 group D member 1
PER	period (gene)
REV-ERBα	reverse orientation c-erbA gene α
RNS	reactive nitrogen species
RORα	retinoic acid receptor-related orphan receptor alpha
ROS	reactive oxygen species
SCN	the suprachiasmatic nucleus
SOD	superoxide dismutase
TNF-α	tumor necrosis factor-alpha
TRE	time-restricted eating
TTFL	transcriptional–translational feedback loop
VSMC	vascular smooth muscle cell
